# Eating Disorder Risk Among Italian University Students: A Cross-Sectional Screening Study Using BMI, EAT-26, and EDE-Q 6.0

**DOI:** 10.3390/nu18121984

**Published:** 2026-06-19

**Authors:** Valeria Gosti, Antonella Coletta, Andrea Carolina Vinci, Francesca Massaro, Francesca Foti, Giacomo Koch, Francesca Gelfo, Viviana Betti, Laura Petrosini, Silvia Picazio

**Affiliations:** 1 Department of Human Sciences, Università degli Studi Guglielmo Marconi, 00193 Rome, Italy; v.gosti@unimarconi.it (V.G.); s.picazio@hsantalucia.it (S.P.); 2 Santa Lucia Foundation (Scientific Research and Treatment Institute), 00179 Rome, Italy; 3 Department of Educational Sciences, University of Catania, 95124 Catania, Italy; 4 Department of Neuroscience and Rehabilitation, University of Ferrara, 44121 Ferrara, Italy; 5 Department of Psychology, Sapienza University of Rome, 00185 Rome, Italy

**Keywords:** eating disorders, body mass index, EAT-26, EDE-Q 6.0, gender differences, university students

## Abstract

**Background/Objectives:** Eating disorders (EDs) are among the most severe psychiatric conditions affecting young people, with increasing prevalence in the post-pandemic period. This study assessed the prevalence of ED risk and dysfunctional eating behaviors among Italian university students, a population poorly characterized with respect to ED risk, and examined associations with key socio-demographic and anthropometric variables. **Methods**: A cross-sectional online screening study was conducted between August 2023 and February 2026 with 401 Italian university students (306 women and 95 men). Participants completed the validated Italian versions of the Eating Attitudes Test-26 (EAT-26) and the Eating Disorder Examination Questionnaire 6.0 (EDE-Q 6.0), alongside self-reported anthropometric data. Multiple linear regression analyses were performed to identify predictors of ED risk scores. **Results:** A total of 37.9% of participants had BMI outside the normal range (19.7% underweight; 18.2% overweight or obese). EAT-26 scores exceeded the clinical cut-off in 28.4% of participants (women: 35.6%; men: 5.3%). EDE-Q 6.0 global scores exceeded the clinical cut-off in 21.0% (women: 25.8%; men: 5.3%). Only 45.4% showed no anthropometric or questionnaire-based screening risk indicators (i.e., scores above the clinical cut-off on the EAT-26 or EDE-Q 6.0). BMI was negatively associated with EAT-26 scores in the total sample and in women, while a positive association between BMI and EDE-Q 6.0 scores was observed in men. **Conclusions**: A substantial proportion of Italian university students, particularly women, presented screening-based indicators of ED risk. The combined use of anthropometric and questionnaire-based screening tools provides a more comprehensive risk assessment than either measure alone, highlighting the need for multidimensional screening programs.

## 1. Introduction

Eating disorders (EDs) are characterized by a multifactorial etiology and a tendency toward chronic progression, representing a serious public health concern for adolescents and young people in Western countries [1,2,3,4]. These disorders are associated with severe psychophysical consequences; in particular, anorexia nervosa is recognized as the psychiatric condition with the highest mortality rate [5,6,7].

In recent years, and particularly since the onset of the COVID-19 pandemic, the incidence of EDs has increased considerably, especially among women [8,9,10]. In Italy, a sharp rise in hospital admissions for EDs has been recorded, with a marked increase among adolescents [8,11,12]. Eating disorders themselves are now described as a growing global epidemic [7], with an increasingly early age of onset [5,7] and prevalence rates reaching up to 15% in females and 10% in males during adolescence [4,5,7]. A large-scale retrospective study conducted on more than 46 million participants aged under 27 years documented a nearly eightfold increase in prevalence between 2017 and 2022, from 120 to 916 cases per 100,000 people [8,13]. In Europe, Italy has a very high prevalence (1008.65/100,000), preceded only by Spain (1104.85/100,000) and followed by Finland (929.61/100,000), Sweden (927.22/100,000), and Austria (911.26/100,000) [8,14]. A post-pandemic Italian study reported a prevalence of 31.0% of adolescents at risk for EDs, in a representative sample of 6551 high school students [15]. Eating disorders represent a significant and growing public health concern worldwide. Global burden estimates indicate a substantial prevalence across populations; however, their true prevalence is likely underestimated due to underdiagnosis and methodological limitations in epidemiological assessment [16]. However, monitoring systems rely predominantly on anthropometric indicators such as Body Mass Index (BMI), which do not account for the psychological and behavioral dimensions central to the diagnosis of eating disorders, leaving a gap in the detection of subclinical risk profiles. A recent study using dual-energy X-ray absorptiometry found that BMI overestimates the prevalence of underweight and overweight in more than one-third of individuals, despite being reliable in the normal-weight range [17]. BMI is therefore an approximate anthropometric index, which does not distinguish between fat mass and lean mass and may lead to misclassifications, especially in the presence of atypical body compositions [18]. Body composition is influenced by factors such as ethnicity, sex, and metabolic conditions, which BMI cannot account for [19]. These outcomes highlight the complexity of the relationship between BMI and eating psychopathology and reinforce the need for a multidimensional approach that integrates validated psychometric tools with anthropometric measures. However, it should be acknowledged that both BMI-based and self-reported screening approaches carry inherent limitations, including potential misclassification, reporting bias, and the inability to capture the full clinical complexity of eating disorders. These constraints underscore the importance of interpreting screening results with appropriate caution.

Notably, few studies to date have evaluated the risk of eating disorders with a focus on Italian university students. Evaluating the vulnerability of Italian university students to this risk is important for assessing the problem’s magnitude and developing targeted prevention, research, and treatment programs. The university period is characterized by a convergence of psychological, social, and behavioral transitions, including increased academic pressure, changes in living conditions, greater autonomy in food choices, and greater exposure to body image ideals [20,21,22,23]. Consistent associations between academic stress, emotional distress, body dissatisfaction, and dysfunctional eating behaviors have been documented [22,24,25], suggesting that the university environment may be a critical context for the emergence or consolidation of disordered eating patterns. For example, a recent study among first-year university students in Sweden reported a prevalence of eating disorders of 28% [26].

Eating disorders rarely manifest abruptly; on the contrary, their onset is usually preceded by a series of subthreshold symptoms and dysfunctional behaviors that can be considered precursors of the full clinical condition [27]. Early identification of such risk profiles, therefore, may represent a fundamental strategy for public health prevention and intervention.

The present study aimed to assess the risk of eating disorders among Italian university students through an online screening campaign using BMI and two validated questionnaire-based tools to evaluate eating behavior. We examined associations between eating disorder risk and key sociodemographic variables to identify potentially vulnerable groups. Based on available epidemiological evidence, we expected that a large proportion of Italian university students, particularly women, would present a high risk.

Beyond the primary screening objective, participants who provided contact details received personalized feedback on their results as a subsequent step of the campaign, aimed at raising awareness of early indicators of dysfunctional eating behaviors and promoting health and well-being.

## 2. Materials and Methods

### 2.1. Study Design and Procedure

This study was conducted between August 2023 and February 2026, recruiting a sample of Italian university students. Participants were recruited via QR-coded posters, university social media channels, and instant messaging groups distributed across different regions of Italy. Data were collected using Google Forms (version N/A, Google LLC, Mountain View, (CA), United States. Prior to completing the survey, participants were provided with information regarding the study objectives, data handling, and privacy policy, and electronic informed consent was obtained. Participants were then asked to provide a telephone number and email address to receive personalized feedback on their eating behaviors. The survey collected demographic and anthropometric information, including age, height, weight, and city of residence, alongside two standardized screening instruments: the Eating Attitudes Test-26 (EAT-26) [28,29] and the Eating Disorder Examination Questionnaire (EDE-Q 6.0) [30,31].

### 2.2. Participants

A total of 504 responses to the online survey were collected. The inclusion criteria were: (1) voluntary participation with full completion of the questionnaire and (2) current enrolment in a university degree program. The exclusion criteria were: (1) age below 18 years and (2) absence of consent for data processing. The study protocol complied with the Declaration of Helsinki and was approved by the Santa Lucia Foundation IRCCS of Rome Ethics Committee (Protocol number: CE/PROG.885 and date of approval: 1 June 2022).

### 2.3. Clinical Risk Assessment of Eating Disorders

#### 2.3.1. Anthropometric Data

The Body Mass Index (BMI; kg/m^2^) was calculated from self-reported height and weight. It should be noted that reliance on self-reported anthropometric data may introduce reporting bias, particularly in a population sensitive to body image, and may reduce the accuracy of BMI-based classifications. According to the World Health Organization (WHO) classification, participants were categorized as underweight (BMI < 18.5), normal weight (BMI 18.5–24.99), overweight (BMI 25.0–29.99), or obese (BMI ≥ 30.0).

#### 2.3.2. Eating Behavior Questionnaires

Eating disorder risk was assessed using the Eating Attitudes Test-26 (EAT-26) [28], a widely used standardized screening tool. The validated Italian version was used in the present study [29]. The EAT-26 comprises 26 items organized into three subscales: Dieting, Bulimia and Food Preoccupation, and Oral Control. Responses are provided on a six-point Likert scale ranging from ‘never’ to ‘always’. Each item (except item 26) is scored from 0 to 3 (‘always’ = 3, ‘almost always’ = 2, ‘often’ = 1; ‘seldom’, ‘rarely’, and ‘never’ = 0). Item 26 is reverse-scored. Total scores range from 0 to 78, with scores ≥ 20 indicating clinical risk for an eating disorder and higher scores reflecting greater symptom severity.

Eating disorder symptoms were also assessed using the Eating Disorder Examination Questionnaire (EDE-Q 6.0) [30] in its validated Italian version [31]. This self-administered tool comprises 28 items that measure the frequency and severity of eating disorder symptoms over the previous four weeks on a seven-point Likert scale. The EDE-Q 6.0 yields four subscale scores, Restraint, Eating Concern, Weight Concern, and Shape Concern, and a global score reflecting the overall severity of eating psychopathology. Subscale cut-off scores are as follows: Restraint ≥ 3.89; Eating Concern ≥ 2.34; Weight Concern ≥ 4.32; Shape Concern ≥ 5.35; and Global score ≥ 3.98. Scores above these thresholds indicate, at the screening level, a potential risk of disordered eating behaviors [30,31].

### 2.4. Descriptive Analyses

Descriptive statistics were computed for all variables. Continuous variables were expressed as mean ± standard deviation; categorical variables were reported as frequencies and percentages. To provide an overview of the distribution of risk profiles within the sample, participants were classified by BMI and their EAT-26 and EDE-Q 6.0 scores.

Participants were classified into one of five categories based on their BMI and their scores on the EAT-26 and EDE-Q 6.0, according to the criteria established by a clinical psychologist expert in the diagnosis of eating disorders (Silvia Picazio). These categories are intended as exploratory, descriptive screening classifications and do not reflect validated diagnostic groupings or clinical subtypes. In cases where participants met criteria for more than one category, a hierarchical classification procedure was applied, prioritizing questionnaire-based screening status (EAT-26 or EDE-Q 6.0 score above vs. below the clinical cut-off) as the primary determinant of risk allocation, followed by BMI-based stratification to assign participants to the most appropriate subgroup.

Specifically: (1) Restrictive-Screening Risk (RR)—BMI < 20 and EAT-26/EDE-Q 6.0 scores above the cut-off; (2) Healthy Participants (HP)—BMI 18.5–26.5 and EAT-26/EDE-Q 6.0 scores below the cut-off; (3) Bulimic-Screening Risk (BR)—BMI ≥ 24 and EAT-26/EDE-Q 6.0 scores above the cut-off; (4) Altered BMI (AB)—BMI ≤ 18.5 or ≥ 26.5 and EAT-26/EDE-Q 6.0 scores below the cut-off; (5) Altered Testing (AT)—BMI 20–23 and EAT-26/EDE-Q 6.0 scores above the cut-off. In cases where BMI ranges overlapped (18.5–20 kg/m^2^), group allocation was determined primarily by questionnaire-based screening criteria (EAT-26; EDE-Q 6.0), with participants exceeding clinical cut-off scores assigned to the RR group, whereas participants below cut-off scores were classified as HP.

BMI thresholds were intentionally selected to allow exploratory stratification of weight-related profiles, extending partially beyond conventional categories proposed by the World Health Organization and clinical cut-offs, taking into account more ecologically representative conditions. It is important to note that the questionnaires’ cut-off scores were used as screening indicators rather than diagnostic criteria, in line with their intended use. Therefore, the resulting categories should be considered purely descriptive, providing a preliminary overview of potential risk patterns. They do not reflect validated diagnostic groupings, nor are they derived from established clinical classification systems.

### 2.5. Statistical Analyses

Statistical analyses were conducted using Jamovi (version 2.7.17). Two multiple linear regression models were performed: one with the total EAT-26 score as the outcome and one with the total EDE-Q 6.0 score. In both models, eating disorder risk was operationalized as a continuous total score, and BMI, age, and gender were entered as independent variables. Given the gender imbalance in the sample (76.3% women), additional regression analyses were conducted separately for women and men for each outcome measure to reduce potential bias arising from unequal group representation and to explore whether the observed associations differed by gender. Statistical significance was set at *p* < 0.05.

## 3. Results

### 3.1. Demographic Characteristics of Participants

Of the 504 responses, 103 participants were excluded: 67 were not university students, and 36 had not provided consent for data processing. This yielded a final sample of 401 university students, of whom 306 (76.3%) were women and 95 (23.7%) were men (Table 1). The mean age of participants was 23.2 ± 6.3 years (age range: 18–65 years). Mean height was 1.70 ± 0.09 m and mean weight was 61.5 ± 13.8 kg, yielding a mean BMI of 21.78 ± 4.22 kg/m^2^. BMI could not be calculated for 2 participants (0.5%) who did not report their weight; however, these individuals were retained in the sample because their EAT-26 and EDE-Q 6.0 scores were above the clinical cut-off for eating disorder risk. According to WHO guidelines, 61.6% of participants were of normal weight, 19.7% were underweight, and 18.2% were in the overweight or obese range (14.7% overweight and 3.5% obese). The sample was distributed across three geographical areas of Italy: Northern Italy (8.2%), Central Italy (82.1%), and Southern Italy (9.7%) (Table 1).

### 3.2. Clinical Risk Assessment of Eating Disorders

#### 3.2.1. Eating Attitudes Test-26 (EAT-26)

The mean total EAT-26 score for the overall sample was 15.1 ± 15.6, with 114 participants (28.4%) scoring at or above the clinical cut-off of ≥20 (Table 2). Across both the total sample and when stratified by sex, the Dieting subscale consistently yielded the highest mean scores.

#### 3.2.2. Eating Disorder Examination Questionnaire 6.0 (EDE-Q 6.0)

The mean total EDE-Q 6.0 score for the overall sample was 2.29 ± 1.66, with 84 participants (21.0%) scoring at or above the clinical cut-off of ≥ 3.98. The proportions of participants exceeding the respective subscale cut-offs were: Eating Concern, 28.2% (cut-off ≥ 2.34); Weight Concern, 25.7% (cut-off ≥ 4.32); Shape Concern, 18.0% (cut-off ≥ 5.35); and Restraint, 17.2% (cut-off ≥ 3.89) (Table 3).

### 3.3. Descriptive Risk Categorization Based on BMI and Clinical Scores

**Figure 1:**
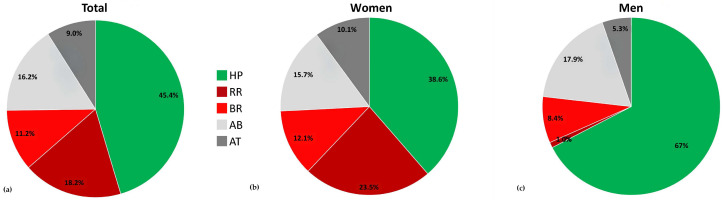
Distribution of participants across operational risk categories based on BMI and EAT-26/EDE-Q 6.0 scores. Operational categories: Healthy Participants (HP), BMI 18.5–26.5 and scores below cut-off; Restrictive-Screening Risk (RR), BMI < 20 and scores above cut-off; Bulimic- Screening Risk (BR), BMI ≥ 24 and scores above cut-off; Altered BMI (AB), BMI ≤ 18.5 or ≥ 26.5 and scores below cut-off; Altered Testing (AT), BMI 20–23 and scores above cut-off. These categories represent operational classifications for descriptive purposes only and do not correspond to formal clinical diagnoses. (**a**) Total sample (n = 401); (**b**) Women (n = 306); (**c**) Men (n = 95; percentages may not total 100 due to rounding).

Based on the exploratory screening criteria described in Section 2.4., 45.4% of participants fell into the HP, 18.2% into the RR, 11.2% into the BR, 16.2% into the AB, and 9.0% into the AT patterns (Figure 1a).

Given the marked gender imbalance in the sample (306 women and 95 men), the risk categorization was also examined separately by sex. Among women, 38.6% were classified as HP, 23.5% as RR, 12.1% as BR, 15.7% as AB, and 10.1% as AT (Figure 1b). Among men, 67.0% were classified as HP, 1.0% as RR, 8.4% as BR, 17.9% as AB, and 5.3% as AT (Figure 1c). It should be noted that these categories represent purely descriptive and exploratory screening classifications based on operational criteria and should not be interpreted as clinical diagnoses or validated diagnostic groupings.

### 3.4. Multivariate Regression Analyses

**Figure 2:**
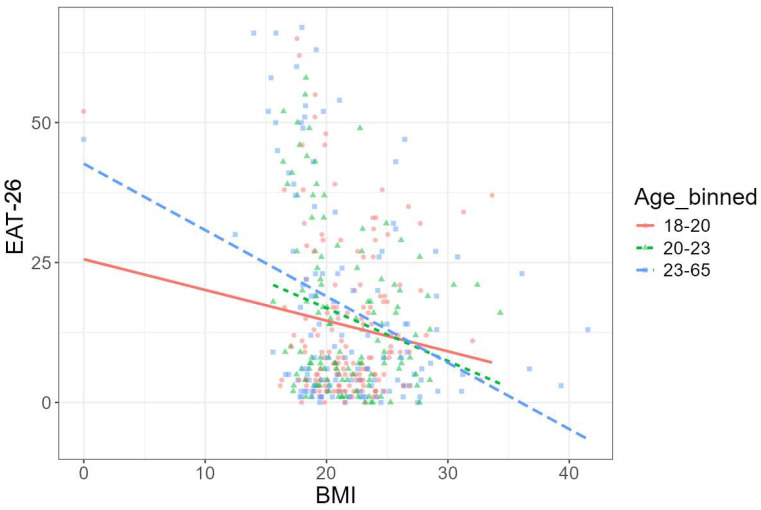
Scatter plot of the association between BMI and EAT-26 total scores, stratified by age group (18–20, 20–23, and 23–65 years). Regression lines are displayed for each subgroup. A significant inverse association between BMI and EAT-26 scores was observed, indicating that higher BMI was associated with lower EAT-26 scores. The steeper slope of the regression line in older age groups indicates a stronger inverse association between BMI and EAT-26 scores with increasing age.

**Figure 3:**
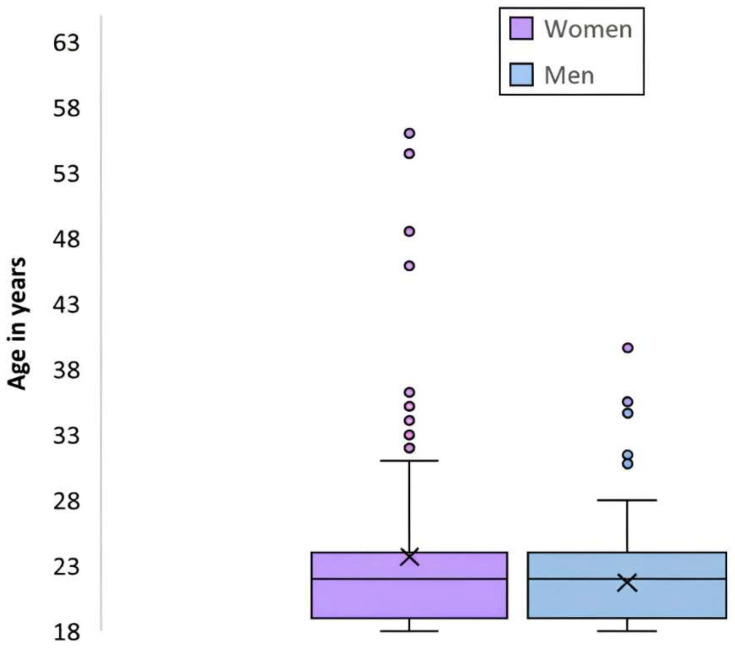
Box plot of the age distribution of the study sample. The horizontal line within the box indicates the median; the marker indicates the mean; the box spans the interquartile range (Q1–Q3); whiskers extend to 1.5 × IQR. Points beyond the whiskers represent participants whose age falls outside the interquartile range. A small number of participants (16 women and 1 man) reported an age above 30 years.

The results of the multiple linear regression analyses are presented in Table 4. For EAT-26 as the dependent variable, the model explained approximately 14% of the variance in total scores (R^2^ = 0.140). BMI showed a significant negative association with EAT-26 scores (β = −0.699; *p* < 0.001), indicating that higher EAT-26 scores were associated with lower BMI. Age showed a significant positive association (β = 0.365; *p* = 0.002), indicating that higher EAT-26 scores were associated with higher age (Figure 2); this result should be interpreted in light of the age distribution of the sample, which was concentrated between 19 and 25 years (Figure 3). Gender was the strongest predictor (β = 8.037; *p* < 0.001): women scored approximately 8 points higher than men on average, though this finding should be interpreted in light of the marked gender imbalance in the present sample.

A separate regression model was fitted to predict total EDE-Q 6.0 scores using the same predictors (R^2^ = 0.104), accounting for approximately 10.4% of the variance. Gender was again a significant predictor (β = 1.278; *p* < 0.001), with women scoring approximately 1 point higher than men on the EDE-Q 6.0. Neither age (β = 0.014; *p* = 0.271) nor BMI (β = 0.035; *p* = 0.080) reached statistical significance. Given the modest proportion of variance explained by both models, the present results should be interpreted as exploratory associations rather than strong predictive or explanatory models of eating disorder risk.

#### 3.4.1. Multivariate Regression Analyses in Female Participants

**Figure 4:**
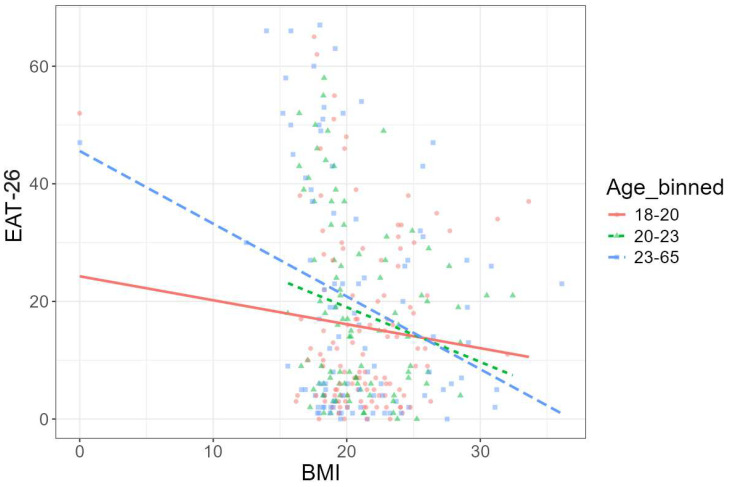
Scatter plot of the association between BMI and EAT-26 total scores in women (n = 306), stratified by age group (18–20, 20–23, and 23–65 years). Regression lines are displayed for each subgroup. Consistent with the results for the total sample, a significant inverse association between BMI and EAT-26 scores was observed. The steeper slopes of the regression lines in older age groups (blue and green dashed lines) compared to the youngest age group (red continuous line) indicate a stronger inverse association between BMI and EAT-26 scores with increasing age.

In the women subsample (n = 306), the regression model for EAT-26 scores yielded an R^2^ of 0.074, indicating that approximately 7.4% of the variance in EAT-26 scores was explained by the predictors. Age again showed a significant positive association (β = 0.383; *p* = 0.004), and BMI showed a significant negative association (β = −0.947; *p* < 0.001), indicating that higher EAT-26 scores were associated with lower BMI (Figure 4).

For the EDE-Q 6.0 in women, the model explained only 0.6% of the variance (R^2^ = 0.006). None of the included predictors were statistically significant (Table 4).

#### 3.4.2. Multivariate Regression Analyses in Male Participants

In the men subsample (n = 95), the regression model for EAT-26 scores explained approximately 1.4% of the variance (R^2^ = 0.014). Neither age (β = −0.042; *p* = 0.840) nor BMI (β = 0.178; *p* = 0.260) reached statistical significance.

**Figure 5:**
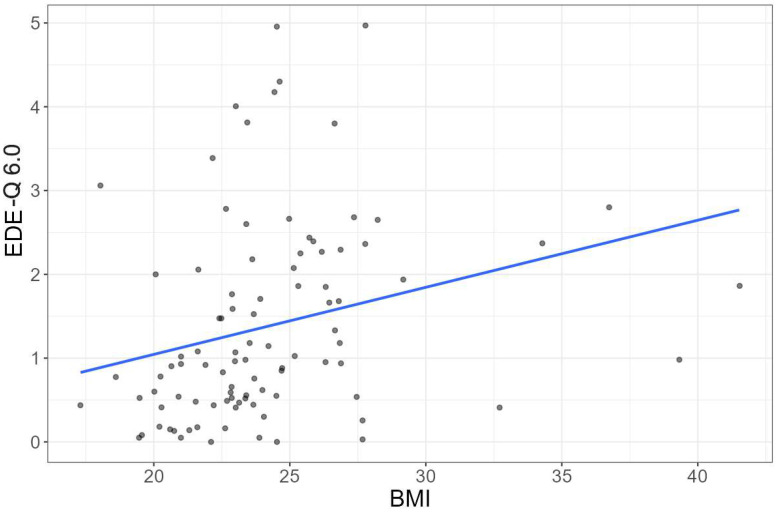
Association between BMI and EDE-Q 6.0 total scores in men (n = 95), with a fitted regression line. Each dot represents a participant, while the blue line represents the fitted linear regression model. BMI was significantly and positively associated with EDE-Q 6.0 total scores.

For the EDE-Q 6.0 in men, the model explained approximately 7.4% of the variance (R^2^ = 0.074). BMI showed a significant positive association (β = 0.078; *p* = 0.012), indicating that higher EDE-Q 6.0 scores were associated with higher BMI (Figure 5). Age was not a significant predictor (β = 0.017; *p* = 0.680) (Table 4).

## 4. Discussion

The present study aimed to assess the risk of eating disorders among Italian university students using a multidimensional approach that combined anthropometric data (BMI) with two validated questionnaire-based screening tools (EAT-26 and EDE-Q 6.0). The main findings indicate that a substantial proportion of participants exhibited risk indicators on at least one screening dimension: 37.9% had a BMI outside the normal range, 28.4% exceeded the EAT-26 clinical cut-off, and 21.0% exceeded the EDE-Q 6.0 global cut-off. Gender was the strongest predictor in both regression models, with women showing markedly higher risk scores. However, these associations should be interpreted with caution, as the cross-sectional design does not allow causal inferences or the establishment of temporal relationships. Overall, only 45.4% of participants showed no anthropometric or questionnaire-based screening risk indicators.

Specifically, the present findings indicate that a significant proportion of Italian university students show anthropometric profiles associated with potential nutritional risk. According to the WHO classification, 37.9% of the sample had an altered BMI: 19.7% were underweight, and 18.2% fell within the overweight/obese range. By comparison, according to ISTAT (2021) [32], 2.9% of the adult population is underweight; the most recent ISTAT report (2023) [33] indicates that 46.9% is overweight or obese.

In the present university sample, the underweight category is therefore more represented than the national ISTAT figure, but it is consistent with the well-documented vulnerability of young people in higher education to restrictive eating behaviors [34,35]. This discrepancy is further supported by Eurostat data indicating that the 16–24 age group has the lowest proportion of overweight individuals in the EU (20.3%) compared to 50.6% across all adult age groups [36], and by a study on 734 Italian university students reporting 5.6% of women in the underweight category [37]. Overall, the BMI distribution in our sample is consistent with that observed in other university populations, both Western and non-Western. Okasha et al. [38] observed higher critical BMI values in 319 university students in Cairo, with 35.8% of medical students and 24.7% of non-medical students at risk for eating disorders. Kwilosz et al. [39] reported a prevalence of 24.02% overweight and 10.24% obesity in Polish students. Finally, Alzahrani et al. [40], in 404 Saudi students, identified gender differences in BMI, with greater underweight in women (11% vs. 2.4%) and greater overweight/obesity in men (59% vs. 40%), and an overall distribution of 7.2% underweight and 18.2% obesity. Taken together, these data confirm that the university population has a higher rate of nutritional risk profile than the general adult population, in line with international evidence. Caution is nonetheless required when interpreting BMI-based results in the context of eating disorder research. Alterations in BMI may arise from various medical and behavioral conditions not necessarily related to EDs, making it an insufficient standalone diagnostic indicator and, at best, a general screening tool [41,42,43,44]. Furthermore, BMI is unable to capture the psychological dimensions central to the diagnosis of EDs, such as distorted body image, weight-control behaviors, and associated emotional distress, as defined in the diagnostic criteria of the DSM-5 and ICD-11 [45,46]. Recent evidence suggests that dysfunctional eating behaviors and body dissatisfaction represent more direct determinants of risk than the BMI alone [47].

In the present study, when ED risk was assessed using the EAT-26, 28.4% of the sample exceeded the clinical cut-off (≥20). This value is higher than the 19.7% prevalence reported in a meta-analysis of university populations [48], but it could be consistent with the increased risk observed in the post-pandemic period, as we recruited participants from 2023 to 2026. In support of this interpretation, a large-scale longitudinal study conducted in the United States reported an increase in eating disorder symptomatology among university students between the pre-pandemic and pandemic periods [49]. Similar findings have been reported in Norway, where an increase in self-reported eating disorder symptoms and diagnoses was observed among university students in recent years [50]. The gender differences observed in the present sample, with a higher risk prevalence among women (35.6%) compared to men (5.3%), are consistent with established epidemiological evidence [22,26,34,35,39,51].

A further relevant aspect concerns the discrepancy between anthropometric and questionnaire-based screening results: while 37.9% of participants had an altered BMI, only 28.4% exceeded the EAT-26 clinical cut-off. This suggests that a non-negligible proportion of the sample presents weight alterations not necessarily associated with eating psychopathology. In quantitative terms, approximately 9.5% of participants may be identified as at risk according to BMI for reasons not directly related to an eating disorder, reinforcing the need to integrate specific questionnaire-based screening tools.

The global EDE-Q 6.0 score exceeded the clinical cut-off in 21.0% of participants, with a clear gender difference (25.8% of women vs. 5.3% of men). The EDE-Q 6.0, therefore, appears more stringent than the EAT-26 in identifying clinical-level risk, with a prevalence gap of approximately 7 percentage points between the two instruments. This is consistent with the findings from studies conducted in Western Asia, where EAT-26-based prevalence estimates (22.1%) were considerably higher than those based on the EDE-Q (8.0%) [48]. It might be hypothesized that the EAT-26 is more sensitive in detecting dysfunctional eating behaviors, while the EDE-Q 6.0 is more specific in assessing clinical risk; however, rather than implying the superiority of one instrument over the other, these findings suggest that the two tools may assess similar but partially distinct constructs, reflecting complementary dimensions of disordered eating risk. A study comparing the two instruments for assessing eating attitudes appears to confirm our hypothesis that the EDE-Q 6.0 exhibits moderate-to-high sensitivity and specificity in male populations and tends to be less reliable among women [52].

Among the EDE-Q subscales, the highest rates were observed for Eating concern (28.2%) and Weight concern (25.7%), followed by Shape concern (18.0%) and Restraint (17.2%). These findings indicate that the cognitive and emotional dimensions of the relationship with body and food represent central aspects of the observed risk, in line with the literature [38,53,54].

The use of the EDE-Q in prevalence studies remains relatively limited compared to the EAT-26. The systematic review by Alsheweir et al. [55] indicates that most studies use screening tools such as the EAT-26, while only a small number adopt the EDE-Q, suggesting its less frequent use in large-scale studies in student populations.

The integrated risk classification, based on BMI, EAT-26, and EDE-Q 6.0, allowed a more detailed characterization of risk profiles. Only 45.4% of participants were deemed not at risk according to the study’s screening criteria (Figure 1a, HP), while 29.4% fell into author-defined screening-based risk categories (Figure 1a, RR+BR) and 25.2% presented potential screening-based risk profiles (Figure 1a, AT+AB). The gender-stratified analysis confirmed that women had substantially higher rates of RR (23.5% vs. 1.0%) and AT (10.1% vs. 5.3%), whereas men had a higher percentage of altered BMI without test-alteration risk (17.9% vs. 15.7%). Overall, 54.6% of the sample did not fall into a condition free of any anthropometric or questionnaire-based screening risk indicator. These operational categories are not intended as clinical diagnoses and must be interpreted strictly as exploratory descriptive classifications without validated diagnostic value; as such, they should not be used to draw conclusions about the clinical status of the participants but may provide a preliminary framework for identifying subgroups that could benefit from further assessment.

Regression analyses confirmed gender as the strongest predictor of risk. BMI showed different associations in the two sexes: negative with the EAT-26 in women and positive with the EDE-Q in men. These results may reflect a greater relevance of weight-related concerns in men with higher BMI, in line with the literature on male body dissatisfaction [56], although the small size of the male subsample warrants caution. Consistent with this, recent evidence also shows higher rates of underweight classification among women and a greater prevalence of overweight/obesity among men in university samples [40].

Age emerged as a positive predictor of EAT-26 scores in both the total sample and the female subsample, indicating that older students reported higher levels of dysfunctional eating attitudes. This result should be interpreted in light of the age distribution of the present sample, strongly concentrated between 19 and 25 years, with only a small number of participants over 30 years of age (16 women and 1 man; Figure 3). The age effect may therefore not reflect a linear developmental increase in vulnerability to eating disorders, but rather specific stress factors associated with more advanced stages of academic training, such as thesis preparation [51,57,58,59].

Taken together, the present findings support the importance of a multidimensional approach to eating disorder risk screening in university populations. The complementary use of anthropometric and questionnaire-based screening tools enabled a more comprehensive risk characterization than a single approach could achieve. Furthermore, the provision of personalized feedback to all participants represents an innovative element that may represent a potentially valuable component for promoting awareness and early help-seeking, in line with evidence-based stepped-care principles, in which early risk identification is associated with proportionate support. It should be noted, however, that no intervention outcomes were assessed in the present study; therefore, the feedback component should be considered a potential preventive element rather than an evidence-based intervention [60].

The growing public health burden represented by eating disorders, especially in the post-pandemic period, makes the university environment a critical and still resource-poor context for preventive strategies. As documented in a 12-year French longitudinal study, the prevalence of EDs among university students remained stable between 2009 and 2018, before rising sharply in 2021 [61]. A systematic review further confirmed an increase in disordered eating behaviors associated with COVID-19, attributed to greater social isolation, emotional distress, and intolerance of uncertainty [62]. In this context, the present study helps fill a gap in the Italian landscape by providing prevalence data from a large sample of university students, a population that remains understudied compared to adolescents.

### Limitations and Future Directions

Recruitment via online self-selection might have introduced a *selection bias*, since students already sensitized to eating behaviors may have been more likely to participate, thereby substantially inflating apparent risk prevalence estimates. Furthermore, weight and height were self-reported rather than measured directly, introducing a potential *reporting bias*, especially in a population attentive to body image. The marked gender imbalance in the present sample may have introduced a *gender bias* into the regression models. Moreover, because this was a cross-sectional study, any score associated with low symptom frequency would be indistinguishable from one associated with an actual eating disorder risk, leading to overestimation of the risk. Furthermore, although associations emerged between BMI, age, gender, and eating disorder risk, the cross-sectional design has not allowed for establishing any causal inference. Finally, the marked geographic concentration of the sample, with 82.1% of participants recruited from Central Italy, substantially limits the generalizability of the findings to other Italian regions and to the broader Italian university population.

On this basis, future studies should aim to replicate these findings in more representative samples with respect to geography and gender, using directly measured anthropometric data and longitudinal designs. Lastly, since some of the eating behavior may be dictated by the responsibility of providing for oneself, it would be interesting to stratify the students as day-scholars (i.e., those still living at home under parental care) versus those staying on their own, with greater autonomy in food choices. This would be important in determining whether the discrepancy/attitude towards eating habits reflects adjustment to living independently.

## 5. Conclusions

The present study provides exploratory, descriptive evidence that a notable proportion of the screened sample (54.6%) presented at least one indicator potentially associated with eating disorder risk, as identified through validated screening questionnaires combined with self-reported BMI assessment. This striking figure should nonetheless be interpreted with caution. As an observational study, the present work is intended to support screening rather than clinical diagnosis, and the BMI-by-questionnaire categories proposed here should be regarded as exploratory groupings warranting further validation. The associations emerging from the regression models, while informative, account for a limited share of variance and should therefore be interpreted as indicative. Some features of the sample, including its geographic origin, gender distribution and potential selection bias, also suggest a degree of caution when extending the findings to a broader population. Within these considerations, the results support the value of using multidimensional screening approaches in university settings, which may help identify students at potential risk of eating disorders who could benefit from further clinical evaluation and inform the development of targeted preventive initiatives.

## Figures and Tables

**Table 1:** Demographic and anthropometric characteristics of the study sample.

Variable	Total (n = 401)	Women (n = 306) Men (n = 95)
Age [years], M (SD)	23.2 (6.32)	23.7 (6.9) 21.7 (3.0)
Gender, n (%) ^1^		306 (76.3) 95 (23.7)
Height [m], M (SD)	1.70 (0.09)	1.64 (0.06) 1.77 (0.07)
Weight [kg], M (SD)	61.5 (13.8)	56.8 (9.7) 76.3 (14.5)
BMI category, n (%) ^1^		
Underweight	79 (19.7)	77 (25.1) 2 (2.1)
Normal weight	247 (61.6)	183 (59.8) 64 (67.4)
Overweight	59 (14.7)	35 (11.4) 24 (25.3)
Obese	14 (3.5)	9 (2.9) 5 (5.3)
Geographical area, n (%) ^1^		
Northern Italy	33 (8.2)	28 (9.1) 5 (5.3)
Central Italy	329 (82.1)	249 (81.4) 80 (84.2)
Southern Italy	39 (9.7)	29 (9.5) 10 (10.5)

Note. ^1^ Percentages may not total 100 due to rounding. BMI: Body Mass Index; M: mean; SD: standard deviation.

**Table 2:** EAT-26 total and subscale scores in the total sample and by sex.

Variable	Total (n = 401)	Women (n = 306) Men (n = 95)
Total score, M (±SD)	15.1 (15.6)	17.7 (16.8) 6.7 (5.9)
≥20, n (%) ^1^	114 (28.4)	109 (35.6) 5 (5.3)
**Subscale score**		
Dieting, M (±SD)	9.3 (9.5)	10.8 (10.0) 4.5 (4.7)
Bulimia and Food Preoccupation, M (±SD)	2.8 (3.8)	3.3 (4.0) 1.2 (2.1)
Oral Control, M (±SD)	3.0 (4.2)	3.6 (4.5) 1.1 (1.8)

Note. ^1^ Percentages may not total 100 due to rounding. Clinical cut-off for eating disorder risk: EAT-26 total score ≥ 20. EAT-26: Eating Attitudes Test-26; M: mean; SD: standard deviation.

**Table 3:** EDE-Q 6.0 total and subscale scores in the total sample and by sex.

Variable	Total (n = 401)	Women (n = 306) Men (n = 95)
Total score, M (±SD)	2.29 (±1.66)	2.6 (±1.7) 1.4 (±1.2)
≥3.98, n (%) ^1^	84 (21)	79 (25.8) 5 (5.3)
Restraint, M (±SD)	1.93 (±1.71)	2.1 (±1.8) 1.3 (±1.3)
≥3.89, n (%) ^1^	69 (17.2)	63 (20.6) 6 (6.3)
Eating Concern, M (±SD)	1.53 (±1.61)	1.8 (±1.7) 0.7 (±0.9)
≥2.34, n (%) ^1^	113 (28.2)	104 (33.9) 9 (9.5)
Weight Concern, M (±SD)	2.67 (±1.86)	3.0 (±1.9) 1.6 (±1.4)
≥4.32, n (%) ^1^	103 (25.7)	98 (32) 5 (5.2)
Shape Concern, M (±SD)	3.05 (±1.93)	3.4 (±1.9) 1.9 (±1.6)
≥5.35, n (%) ^1^	72 (18)	68 (22.2) 4 (4.2)

Note. ^1^ Percentages may not total 100 due to rounding. Clinical cut-off scores: Global score ≥ 3.98; Restraint ≥ 3.89; Eating Concern ≥ 2.34; Weight Concern ≥ 4.32; Shape Concern ≥ 5.35. EDE-Q 6.0: Eating Disorder Examination Questionnaire 6.0; M: mean; SD: standard deviation.

**Table 4:** Results of multiple linear regression analyses for EAT-26 and EDE-Q 6.0 total scores.

Model Coefficients: EAT-26					
	R^2^	Predictor	Estimate	t	*p*
**Total (n = 401)**	0.140				
Age		0.365	0.116	3.13	0.002 **
BMI		−0.699	0.182	−3.83	<0.001 **
Gender (1–0)		8.037	1.823	4.41	<0.001 **
**Model Coefficients: EDE-Q 6.0**					
	R^2^	Predictor	Estimate	t	*p*
**Total (n = 401)**	0.104				
Age		0.0139	0.0126	1.102	0.271
BMI		0.0346	0.0197	1.754	0.080
Gender (1–0)		1.2780	0.1973	6.476	<0.001 **
**Model Coefficients: EAT-26**					
	R^2^	Predictor	Estimate	t	*p*
**Women (n = 306)**	0.0738				
Age		0.383	0.133	2.88	0.004 **
BMI		−0.947	0.231	−4.10	<0.001 **
**Model Coefficients: EDE-Q 6.0**					
	R^2^	Predictor	Estimate	t	*p*
**Women (n = 306)**	0.00602				
Age		0.0135	0.0138	0.976	0.330
BMI		0.0216	0.0241	0.896	0.371
**Model Coefficients: EAT-26**					
	R^2^	Predictor	Estimate	t	*p*
**Men (n = 95)**	0.0138				
Age		−0.0419	0.206	−0.203	0.840
BMI		0.1777	0.157	1.133	0.260
**Model Coefficients: EDE-Q 6.0**					
	R^2^	Predictor	Estimate	t	*p*
**Men (n = 95)**	0.0737				
Age		0.0165	0.0400	0.413	0.680
BMI		0.0780	0.0304	2.569	0.012 *

Note. BMI: Body Mass Index; EAT-26: Eating Attitudes Test-26; EDE-Q 6.0: Eating Disorder Examination Questionnaire 6.0; Gender coded as 1 = women, 0 = men. * *p* < 0.05; ** *p* < 0.01.

## Data Availability

The datasets generated and/or analyzed during the current study are available from the corresponding author upon reasonable request.

## Abbreviations

The following abbreviations are used in this manuscript:

AB	Altered BMI
AT	Altered Testing
BMI	Body Mass Index
BR	Bulimic-Screening Risk
COVID-19	Coronavirus Disease 2019
DSM-5	Diagnostic and Statistical Manual of Mental Disorders, Fifth Edition
DXA	Dual-energy X-ray Absorptiometry
EAT-26	Eating Attitudes Test-26
EDE-Q 6.0	Eating Disorder Examination Questionnaire 6.0
EDs	Eating disorders
EU	European Union
GBD	Global Burden of Disease
HP	Healthy Participant
ICD-11	International Classification of Diseases, 11th Revision
IQR	Interquartile Range
ISTAT	Italian National Institute of Statistics
M	Mean
QR	Quick Response (code)
RR	Restrictive-Screening Risk
SD	Standard Deviation
US	United States
W	Women
WHO	World Health Organization

## References

[1] Jaruga-Sękowska S., Staśkiewicz-Bartecka W., Woźniak-Holecka J. (2025). The Impact of Social Media on Eating Disorder Risk and Self-Esteem Among Adolescents and Young Adults: A Psychosocial Analysis in Individuals Aged 16–25. Nutrients.

[2] Lorenzoni V., Casti F., D’Arcangelo G., Balluchi L., Minichilli F., Curzio O., Maestro S. (2025). The Effects of Intensive Residential Treatment for Feeding and Eating Disorders (FEDs) in Adolescence: The Case of an Italian Facility. Nutrients.

[3] Dias R.G., Rech R.R., Halpern R. (2023). Prevalence and Associated Factors of Eating Disorder Symptoms in Adolescents: A Cross-Sectional School-Based Study. BMC Psychiatry.

[4] Silén Y., Keski-Rahkonen A. (2022). Worldwide Prevalence of DSM-5 Eating Disorders Among Young People. Curr. Opin. Psychiatry.

[5] Zuccotti G.V. (2016). Manuale Di Pediatria-La Pratica Clinica.

[6] Mushtaq T., Ashraf S., Hameed H., Irfan A., Shahid M., Kanwal R., Aslam M.A., Shahid H., Koh-E-Noor, Shazly G.A. (2023). Prevalence of Eating Disorders and Their Association with Social Media Addiction Among Youths. Nutrients.

[7] Califano M., Pruccoli J., Cavallino O., Lenzi A., Parmeggiani A. (2025). Psychopathological Comorbidities in Children and Adolescents with Feeding and Eating Disorders: An Italian Clinical Study. Pediatr. Rep..

[8] Barbieri V., Zöbl M., Piccoliori G., Engl A., Hager Von Strobele-Prainsack D., Wiedermann C.J. (2025). Adolescent Eating Disorder Risk in a Bilingual Region: Clinical Prevalence, Screening Challenges and Treatment Gap in South Tyrol, Italy. Nutrients.

[9] Sonne H., Kildegaard H., Strandberg-Larsen K., Rasmussen L., Wesselhoeft R., Bliddal M. (2024). Eating Disorders in Children, Adolescents, and Young Adults During and After the COVID-19 Pandemic: A Danish Nationwide Register-Based Study. Int. J. Eat. Disord..

[10] Pruccoli J., Rosa S., Chiavarino F., Cava M., Gazzano A., Gualandi P., Marino M., Moscano F., Rossi F., Sacrato L. (2024). Feeding and Eating Disorders in Children and Adolescents During the COVID-19 Pandemic: Real-Word Data from an Observational, Naturalistic Study. Minerva Pediatr..

[11] Giacomini G., Elhadidy H.S.M.A., Paladini G., Onorati R., Sciurpa E., Gianino M.M., Borraccino A. (2022). Eating Disorders in Hospitalized School-Aged Children and Adolescents during the COVID-19 Pandemic: A Cross-Sectional Study of Discharge Records in Developmental Ages in Italy. Int. J. Environ. Res. Public. Health.

[12] Cappelletto P., Luca L.D., Taddei B., Taddei S., Nocentini A., Pisano T. (2024). COVID-19 Pandemic Impact among Adolescents with Eating Disorders Referred to Italian Psychiatric Unit. Child. Adoles Psych. Nurs..

[13] Lin B.Y., Moog D., Xie H., Sun C., Deng W.Y., McDaid E., Liebesny K.V., Kablinger A.S., Xu K.Y. (2024). Increasing Prevalence of Eating Disorders in Female Adolescents Compared with Children and Young Adults: An Analysis of Real-time Administrative Data. Gen. Psychiatry.

[14] Liu K., Gao R., Kuang H., Ranbo E., Zhang C., Guo X. (2025). Global, Regional, and National Burdens of Eating Disorder in Adolescents and Young Adults Aged 10–24 Years from 1990 to 2021: A Trend Analysis. J. Affect. Disord..

[15] D’Anna G., Lazzeretti M., Castellini G., Ricca V., Cassioli E., Rossi E., Silvestri C., Voller F. (2022). Risk of Eating Disorders in a Representative Sample of Italian Adolescents: Prevalence and Association with Self-Reported Interpersonal Factors. Eat. Weight Disord..

[16] (2022). GBD 2019 Mental Disorders Collaborators Global, Regional, and National Burden of 12 Mental Disorders in 204 Countries and Territories, 1990–2019: A Systematic Analysis for the Global Burden of Disease Study 2019. Lancet Psychiatry.

[17] Milanese C., Itani L., Cavedon V., El Ghoch M. (2025). The WHO BMI System Misclassifies Weight Status in Adults from the General Population in North Italy: A DXA-Based Assessment Study (18–98 Years). Nutrients.

[18] Rothman K.J. (2008). BMI-related errors in the measurement of obesity. Int. J. Obes..

[19] Ralph A.F., Brennan L., Byrne S., Caldwell B., Farmer J., Hart L.M., Heruc G.A., Maguire S., Piya M.K., Quin J. (2022). Management of Eating Disorders for People with Higher Weight: Clinical Practice Guideline. J. Eat. Disord..

[20] Deliens T., Clarys P., De Bourdeaudhuij I., Deforche B. (2014). Determinants of Eating Behaviour in University Students: A Qualitative Study Using Focus Group Discussions. BMC Public Health.

[21] Barakat S., McLean S.A., Bryant E., Le A., Marks P., Aouad P., Barakat S., Boakes R., Brennan L., National Eating Disorder Research Consortium (2023). Risk Factors for Eating Disorders: Findings from a Rapid Review. J. Eat. Disord..

[22] Amengual-Llofriu M.A., Tauler P., Aguiló A. (2025). Risk of Eating Disorders among University Students and Its Association with Dieting, Weight Control Behavior and Non-Substance Addictions. BMC Public Health.

[23] Aboueldahab A., Vanutelli M.E., D’Addario M., Steca P. (2026). Understanding Food Choices Among University Students: Dietary Identity, Decision-Making Motives, and Contextual Influences. Nutrients.

[24] Aljehani N., Alabdrabalnabi A. (2025). Prevalence and Predictors of Disordered Eating Behaviors: A Cross-Sectional Study across 15 Campuses in the Saudi Electronic University. J. Eat. Disord..

[25] Tavolacci M.P., Grigioni S., Richard L., Meyrignac G., Déchelotte P., Ladner J. (2015). Eating Disorders and Associated Health Risks Among University Students. J. Nutr. Educ. Behav..

[26] Strid C., Lindfors P., Andersson C., Berman A.H. (2025). Eating Disorders and Psychiatric Comorbidity among First-Year University Students in Sweden: Prevalence and Risk Factors. J. Eat. Disord..

[27] Gil M., Weinbach N., Desjardins C.D., Stice E. (2025). Risk Factors That Predict Future Onset of Restricting Versus Binge/Purge Anorexia Nervosa in Women: An Exploratory Study. Eat. Disord..

[28] Garner D.M., Olmsted M.P., Bohr Y., Garfinkel P.E. (1982). The Eating Attitudes Test: Psychometric Features and Clinical Correlates. Psychol. Med..

[29] Dotti A., Lazzari R. (1998). Validation and Reliability of the Italian EAT-26. Eat. Weight Disord..

[30] Fairburn C.G., Beglin S.J. (1994). Assessment of Eating Disorders: Interview or Self-Report Questionnaire?. Int. J. Eat. Disord..

[31] Calugi S., Milanese C., Sartirana M., El Ghoch M., Sartori F., Geccherle E., Coppini A., Franchini C., Dalle Grave R. (2017). The Eating Disorder Examination Questionnaire: Reliability and Validity of the Italian Version. Eat. Weight Disord..

[32] ISTAT, Fattori Di Rischio per La Salute, 2021. https://www.istat.it/tavole-di-dati/fattori-di-rischio-per-la-salute-fumo-obesita-alcol-e-sedentarieta-anno-2021/.

[33] ISTAT, Fumo, Alcol, Eccesso Di Peso e Sedentarietà, 2023. https://www.istat.it/comunicato-stampa/fumo-alcol-eccesso-di-peso-e-sedentarieta-anno-2023/.

[34] Galmiche M., Déchelotte P., Lambert G., Tavolacci M.P. (2019). Prevalence of Eating Disorders over the 2000–2018 Period: A Systematic Literature Review. Am. J. Clin. Nutr..

[35] Devoe D.J., Han A., Anderson A., Katzman D.K., Patten S.B., Soumbasis A., Flanagan J., Paslakis G., Vyver E., Marcoux G. (2023). The Impact of the COVID-19 Pandemic on Eating Disorders: A Systematic Review. Int. J. Eat. Disord..

[36] EUROSTAT (2026). Overweight and Obesity, BMI Statistics. https://ec.europa.eu/eurostat/statistics-explained/SEPDF/cache/12376.pdf.

[37] Zaccagni L., Barbieri D., Gualdi-Russo E. (2014). Body Composition and Physical Activity in Italian University Students. J. Transl. Med..

[38] Okasha T., Mohamed Naguib R., Zaki A., Dessouki N. (2026). Risk of Eating Disorders in Relation to Stress in a Sample of Egyptian Students in Ain-Shams University. J. Eat. Disord..

[39] Kwilosz E., Musijowska M., Badora-Musiał K., Zadarko E., Zadarko-Domaradzka M. (2025). Eating Habits, Physical Activity, Body Composition and Cardiorespiratory Fitness in University Students: A Cross-Sectional Study. Nutrients.

[40] Alzahrani H., Naseeb M., Zagzoog A.M., Malaikah S., Alsulami S., Albajri E. (2026). Eating Attitudes across Body Mass Index Categories in Saudi Arabia: A Cross-Sectional Study. Front. Nutr..

[41] Gutin I. (2018). In BMI We Trust: Reframing the Body Mass Index as a Measure of Health. Soc. Theory Health.

[42] Fletcher I. (2014). Defining an Epidemic: The Body Mass Index in British and US Obesity Research 1960–2000. Sociol. Health Illn..

[43] Nicholls S.G. (2013). Standards and Classification: A Perspective on the ‘Obesity Epidemic’. Soc. Sci. Med..

[44] Timmermans S., Epstein S. (2010). A World of Standards but Not a Standard World: Toward a Sociology of Standards and Standardization. Annu. Rev. Sociol..

[45] American Psychiatric Association (2022). Diagnostic and Statistical Manual of Mental Disorders.

[46] World Health Organization (2019). International Classification of Diseases for Mortality and Morbidity Statistics.

[47] Ispas A.G., Forray A.I., Lacurezeanu A., Petreuș D., Gavrilaș L.I., Cherecheș R.M. (2025). Eating Disorder Risk Among Adolescents: The Influence of Dietary Patterns, Physical Activity, and BMI. Nutrients.

[48] Alhaj O.A., Fekih-Romdhane F., Sweidan D.H., Saif Z., Khudhair M.F., Ghazzawi H., Nadar M.S., Alhajeri S.S., Levine M.P., Jahrami H. (2022). The Prevalence and Risk Factors of Screen-Based Disordered Eating among University Students: A Global Systematic Review, Meta-Analysis, and Meta-Regression. Eat. Weight Disord..

[49] Daly M., Costigan E. (2022). Trends in Eating Disorder Risk among U.S. College Students, 2013–2021. Psychiatry Res..

[50] Jacobsen L.M., Haugan G., Dimitropoulos G., Austin A., Sivertsen B., Braaten T., Bjerkeset O. (2025). “Prevalence of Disordered Eating and Eating Disorders Among Norwegian University Students Before and After the COVID-19 Pandemic, 2018 and 2022: The SHoT Study”. J. Eat. Disord..

[51] Varallo G., Ciaramidaro A., Baldini V., Rubichi S., Scorza M. (2025). Indirect Effects of Body Dissatisfaction in the Association Between Intolerance of Uncertainty and Disordered Eating Attitudes: A Cross-Sectional Study on Italian University Female Students. J. Clin. Med..

[52] Agraib L.M., Al-Shami I., Alkhatib B., Orabi A. (2026). Comparing the Eating Attitudes Test (EAT -26) and Disorder Examination Questionnaire (EDE-Q) as Screening Tools for Eating Disorders among Young Adults: A Population-Specific Analysis. Clin. Nutr. ESPEN.

[53] Chaudhari B. (2017). The Relationship of Eating Disorders Risk with Body Mass Index, Body Image and Self-Esteem among Medical Students. Ann. Med. Health Sci. Res..

[54] Thangaraju S., Karpagalakshmi R., Arumuganathan S., Usaid S., Devi S.S., Sethumadhavan V. (2020). A Cross-Sectional Study on Prevalence of Eating Disorder and Body Image Disturbance among Female Undergraduate Medical Students. J. Ment. Health Hum. Behav..

[55] Alsheweir A., Goyder E., Alnooh G., Caton S.J. (2023). Prevalence of Eating Disorders and Disordered Eating Behaviours amongst Adolescents and Young Adults in Saudi Arabia: A Systematic Review. Nutrients.

[56] Lipson S., Sonneville K. (2017). Eating Disorder Symptoms among Undergraduate and Graduate Students at 12 U.S. Colleges and Universities. Eat. Behav..

[57] De Pasquale C., Sciacca F., Conti D., Pistorio M.L., Hichy Z., Cardullo R.L., Di Nuovo S. (2021). Relations Between Mood States and Eating Behavior During COVID-19 Pandemic in a Sample of Italian College Students. Front. Psychol..

[58] El-Zayat A., Sultan S., Alharthi S., Jamal D., Abdullah A., Albusati N. (2025). The Relationship between Perceived Stress and Emotional Eating Among University Students in Saudi Arabia. Discov. Ment. Health.

[59] Mohd Safri S., Azizi Salleh M.A., Mohd Salleh M.S. (2025). Stress on the Menu: Exploring Eating Behaviors in Stressed University Students. Int. J. Acad. Res. Bus. Soc. Sci..

[60] Richardson L.P., Zhou C., Gersh E., Spielvogle H., Taylor J.A., McCarty C.A. (2019). Effect of Electronic Screening With Personalized Feedback on Adolescent Health Risk Behaviors in a Primary Care Setting: A Randomized Clinical Trial. JAMA Netw. Open.

[61] Tavolacci M.-P., Ladner J., Déchelotte P. (2021). Sharp Increase in Eating Disorders among University Students since the COVID-19 Pandemic. Nutrients.

[62] McLean C.P., Utpala R., Sharp G. (2022). The Impacts of COVID-19 on Eating Disorders and Disordered Eating: A Mixed Studies Systematic Review and Implications. Front. Psychol..

